# Combined Replacement of Fishmeal and Fish Oil by Poultry Byproduct Meal and Mixed Oil: Effects on the Growth Performance, Body Composition, and Muscle Quality of Tiger Puffer

**DOI:** 10.1155/2024/1402602

**Published:** 2024-02-15

**Authors:** Lili Zhao, Lin Li, Feiran Zhang, Peng Li, Yanlu Li, Jian Liu, Yuliang Wei, Mengqing Liang, Qiang Ma, Houguo Xu

**Affiliations:** ^1^State Key Laboratory of Mariculture Biobreeding and Sustainable Goods, Yellow Sea Fisheries Research Institute, Chinese Academy of Fishery Sciences, 106 Nanjing Road, Qingdao 266071, China; ^2^Laboratory for Marine Fisheries Science and Food Production Processes, Laoshan Laboratory, 1 Wenhai Road, Qingdao 266237, China; ^3^Qingdao Aquarium, 2 Laiyang Road, Qingdao 266003, China; ^4^North American Renderers Association, 500 Montgomery Street Suite 310, Alexandria 22314, USA

## Abstract

This study aimed to evaluate the effects of combined replacement of fishmeal (FM) and fish oil (FO) with poultry byproduct meal (PBM) and mixed oil (MO, poultry oil: coconut oil = 1 : 1) on growth performance, body composition and muscle quality of tiger puffer (*Takifugu rubripes*). Fish with an average initial body weight of 14.29 g were selected for the feeding experiment. FM accounting for 0%, 5%, and 10% of the diet was replaced by PBM. For each grade of FM replacement, 5% FO or MO was used as added oil. The six experimental diets were designated as FO-FM, MO-FM, FO-5PBM, MO-5PBM, FO-10PBM, and MO-10PBM, respectively. Each treatment was performed in triplicate with 30 fish per replicate. The feeding period was 45 days. There was no significant difference in growth performance among the groups. Dietary supplementation of both PBM and MO had marginal effects on whole-fish proximate composition, except that dietary MO supplementation significantly increased the liver moisture content. In serum, there were no significant differences in contents of triglyceride, total cholesterol, total bile acid, and protein carbonyl among groups, but the malondialdehyde content was reduced by MO. The fatty acid composition in fish mirrored those in the diets, but the omega-3 sparing effects of saturated and monounsaturated fatty acid in MO can still be observed. Dietary PBM and MO had marginal effects on free amino acid composition and texture of fish muscle, but exerted complicated effects on the muscle volatile flavor compound composition. In conclusion, combined fishmeal (10% of the diet) and fish oil (5% of the diet) replacement with poultry byproduct and mixed oil (poultry oil + coconut oil) had no adverse effects on the growth performance and body proximate composition of farmed tiger puffer. However, these replacements changed the muscle flavor compound profile.

## 1. Introduction

The rapid development of aquaculture requires a huge amount of fishmeal (FM) and fish oil (FO). However, the stable supply of FM and FO is becoming a big challenge. Therefore, searching for suitable and efficient alternative protein and lipid sources has been an urgent task for the aquaculture industry. Numerous studies have been conducted in this research area, demonstrating the great potential of terrestrially sourced ingredients [1–8].

Among the terrestrially sourced ingredients, poultry byproducts, including poultry byproduct meal (PBM), and poultry oil (PO), have been widely used in aquafeeds. Previous studies showed that the PBM can replace 25%–70% of FM in fish feeds: 25% for large yellow croaker (*Larimichthys crocea*) [9] and tench (*Tinca tinca*) [10], 30% for pufferfish (*Takifugu obscurus*) [11] and black sea bream (*Acanthoparus schlegelii*) [12], 50% for Atlantic salmon (*Salmo salar*) [13], black sea turbot (*Psetta maeoticus*) [14], and European eel (*Anguilla anguilla*) [15], 40%–60% for hybrid grouper (*Epinephelus fuscoguttatus* ♀ × *Epinephelus lanceolatus* ♂) [16], and 67% for rainbow trout (*Oncorhynchus mykiss*) [17].

Compared to FM replacement by PBM, PO is able to replace FO at higher levels. It has been suggested that PO can replace 30%–100% of FO in fish feeds without compromising fish growth: 33.3% for rainbow trout [18], 50% for Japanese sea bass (*Lateolabrax japonicus*) [19] and Atlantic salmon [20], 75% for Florida pompano (*Seriola lalandi*) [21] and sablefish (*Anoplopoma fimbria*) [22], and 50%–100% for yellowtail kingfish (*Seriola lalandi*) [23], 100% for gilthead sea bream (*Sparus aurata*) [24], brown trout (*Salmo trutta* L.) [25], barramundi (*Lates calcarifer*) [26], and largemouth bass (*Micropterus salmoides*) [27].

All these results suggest that both PBM and PO have great potential as alternative ingredients in fish feeds. However, most of these studies investigated the efficiency of PBM and PO separately. Very limited studies have investigated the efficacy of combined use of PBM and PO in fish feeds. Farmed fish are supposed to provide long-chain polyunsaturated fatty acids (LC-PUFA), in particular eicosapentaenoic acid (EPA, 20 : 5n-3) and docosahexaenoic acid (DHA, 22 : 6n-3), for human consumers. However, when the farmed fish were fed diets with high levels of terrestrially sourced oils, the LC-PUFA contents usually decrease [28–31]. Therefore, when the FO in fish feeds is replaced by terrestrially sourced oils, how to maintain as high LC-PUFA contents in farmed fish products as possible is of great significance to human consumers [30, 32, 33]. Compared to FO, terrestrially sourced oils usually contain higher levels of saturated and monounsaturated fatty acids (SFA and MUFA, respectively). The SFA and MUFA in terrestrially sourced oils have been reported to have “n-3 LC-PUFA sparing effects” [33–42]. Specifically, for PO, this n-3 LC-PUFA sparing effect has been reported in Murray cod (*Maccullochella peelii peelii*) [39], rainbow trout [43], and Atlantic salmon [44]. However, complete FO replacement by PO compromised the fish growth and health in some species, possibly due to the unbalanced contents of SFA and MUFA in PO, which contains more MUFA than SFA [24]. Specifically, for tiger puffer (*Takifugu rubripes*), our previous studies have shown that this species may have a high capacity to utilize SFA [45]. Coconut oil (CCO) is one of the oils which are the richest in SFA. A mixture of PO with CCO could result in a more balanced profile of SFA and MUFA [46].

The present study aimed to evaluate the efficacy of FM and FO replacement with combined use of PBM and a mixture of PO and CCO. Growth, body composition (in particular fatty acid composition), and muscle quality were measured. Tiger puffer, which is an important aquaculture species in East Asia, was the target fish species of this study. Some studies have revealed the efficacy of some alternative oils such as beef tallow, soybean oil, linseed oil, and rapeseed oil in the diets of tiger puffer [33, 47]. However, few studies have been conducted to screen suitable alternative protein sources for tiger puffer diets. Lim et al. [48] found that replacement of 30% FM with soybean meal did not affect the growth of tiger puffer. Wei et al. [49] even found that replacement of 42.8% FM in low-FM (28% of dry matter) diet with fish protein hydrolysate slightly increased the growth of tiger puffer, although no significant difference was observed.

## 2. Materials and Methods

### 2.1. Experimental Diets

Six isonitrogenous (approximately 48% crude protein) and isolipidic (approximately 7.5% crude lipid) experimental diets were formulated. The FM used in this study was Pollock meal (super level, steamed dried, Tecnologica De Alimentos S.A., Peru) with a protein content of 69.0% and a lipid content of 9.9% (of dry matter). There were three grades of FM replacement with PBM. For these three grades, the FM level was 45%, 40%, and 35% (dry matter basis), respectively, and accordingly the PBM level was 0%, 5%, and 10%, respectively. The PBM supplied by North American Renderers Association (CA, USA) had a protein content of 66.5% and a lipid content of 13.9% (of dry matter). For each grade of FM replacement, 5% FO or mixed oil (MO, PO : CCO = 1 : 1) was used as added oil. The six experimental diets were named FO-FM, MO-FM, FO-5PBM, MO-5PBM, FO-10PBM, and MO-10PBM, respectively. The PO was produced along with the PBM production. The byproducts of chicken processing, mainly including skin, skeleton, trims and viscera, were first boiled and then centrifuged to separate the oils. The formulation and proximate composition of the six experimental diets are presented in Table 1. The fatty acid compositions of FO, MO, and experimental diets are presented in Table 2. The diets were made with a pelleting machine (single-screw, laboratory-level) and dried at 55°C. The diets were stored at a refrigerator room (−20°C) prior to use.

### 2.2. Feeding Procedure and Sampling

Tiger puffer juveniles with an average initial body weight of 14.29 g were used in this feeding experiment. Fish were purchased from Tangshan Haidu Seafood Co., Ltd. (Tangshan, China), and reared in Yellow-Sea Aqua Co., Ltd. (Yantai, Shandong Province, China). To prevent cannibalism, which is common for tiger puffer, the lower fish teeth were cut short before the feeding trial. Before the start of the feeding trial, the experimental fish were temporarily raised in polyethylene tanks (2 m^3^) and fed a commercial feed (protein content, 50% dry matter; lipid content, 8% dry matter; Qingdao Surgreen Biological Engineering Co. Ltd., Qingdao, China) for 2 weeks to acclimate to the experimental conditions. A flow-through seawater (salinity in the range of 28–32) system was used for the feeding experiment. A total of 540 fish were randomly allocated into 18 experimental tanks (0.7 × 0.7 × 0.4 m^3^). Each diet was randomly assigned to triplicate tanks, and each tank had 30 fish. Fish were fed to apparent satiation by hand three times daily (7:30, 12:30, and 18:30). Uneaten feeds were siphoned out and the numbers of uneaten feeds in each tank after each feeding were recorded to adjust the feed consumption data (based on an average weight of pellets). The feeding duration was 45 days. During the whole feeding period, the water temperature ranged from 22 to 28°C; pH in the range of 7.4–7.8; dissolved oxygen >5 mg/L; ammonia-N <0.5 mg/L; and nitrite <0.2 mg/L.

At the end of the feeding trial, fish were first fasted for 24 hr before sampling. The weight and survival of fish in each tank were measured and recorded. After anesthetization with eugenol (eugenol: water = 1/10,000), three fish from each tank were collected for the assay of proximate composition of whole fish. Four more experimental fish from each tank were randomly collected for the collection of the serum, muscle, and liver samples. From each fish, two pieces (2 × 2 cm^2^) of dorsal muscle were collected from each body side. In the following analysis, the muscle samples were then cut into smaller pieces for the assay of muscle texture (can be reused for other assays), fatty acid composition, proximate composition, peroxidation products, free amino acid composition, volatile flavor compound profile, as well as gene expression. A pooled sample from four fish of each tank was used for each assay. Two pieces (2 cm from the small tip) of liver tissues were collected from each fish, for the analysis of fatty acid composition and gene expression. Samples from each tank were also pooled for the analysis. The blood from the caudal vein was collected, and the serum samples were collected as previously described [50]. All protocols of fish rearing and sampling practices in this study, were reviewed and approved by the Animal Care and Use Committee of Yellow Sea Fisheries Research Institute.

### 2.3. Analysis of the Proximate Composition of Fish and Diets

The proximate composition of experimental diets, whole fish, and tissue samples was analyzed with the methods of Association of Official Analytical Chemists. The moisture content was assayed by drying at 110°C. The crude protein content and lipid content were measured with the Kjeldahl (Foss 2300, *N* × 6.25) and Soxhlet method (Foss Soxtec™ 2050, petroleum ether extraction), respectively. The ash content was assayed by incineration at 550°C for 8 hr.

### 2.4. Biochemical Parameters of Serum and Muscle

Serum and muscle samples from four fish of each tank were pooled. The total cholesterol (TC), total bile acid (TBA), malondialdehyde (MDA), total triglyceride (TG), and protein carbonyl (PC) concentrations were analyzed with commercial kits purchased from the Nanjing Jiancheng Bioengineering Institute (Nanjing, Jiangsu Province, China).

### 2.5. Mitochondrial DNA Copy Number

The DNA was extracted from liver and muscle samples with the DP324 kit (Tiangen, Beijing, China). Specific primers target genes (cytochrome B (CYTB) of mitochondrial DNA and 16S rRNA) and reference genes (*β*-actin and ef1*α*) were designed (Table 3). The reaction system of PCR consists of 1 *μ*L cDNA template, 0.4 *μ*L forward primer (10 *μ*M), 0.4 *μ*L reverse primer (10 *μ*M), 5 *μ*L SYBR Green Pro Taq HS Premix II, and 3.2 *μ*L sterilized water. The PCR program was: 95°C for 30 s followed by 40 cycles of “95°C for 5 s, 57°C for 30 s, and 72°C for 30 s”. Other method details can be found in our previous publications [51].

### 2.6. Analysis of Fatty Acid Composition and Free Amino Acid

The fatty acid compositions of oil, diet, muscle, and liver were analyzed with gas chromatography (GC2010 pro, Shimadzu, Kyoto, Japan) equipped with a flame ionization detector and a quartz capillary column (SH-RT−2560, 100 m × 0.25 mm × 0.20 *μ*m). Lipids were first extracted from the samples using the chloroform methanol method. Fatty acids in the lipid samples were then saponified and methylated with boron trifluoride and KOH-methanol. The fatty acid contents are expressed as % total fatty acids (TFA). More details can be found in our previous publications [51].

The muscle samples were deproteinized using trichloroacetic acid (6%) and centrifuged at 10,000*g* at 4°C for 10 min to obtain the supernatant. Amino acid contents were determined using the L-8900 amino acid analyzer (Hitachi, Japan).

### 2.7. Analysis of Volatile Organic Compounds in the Muscle

Muscle samples from three typical groups, FO-FM, MO-FM, and FO-10PBM, were used for the volatile organic compounds analysis. The comparison between groups FO-FM and MO-FM and that between groups FO-FM and FO-10PBM most typically indicate the influence of dietary MO and PBM, respectively. This analysis was conducted with gas chromatography-ion migration spectrometry (GC-IMS). A FlavourSpec® platform (G.A.S, Dordmund, Germany) and a MXT-5 column (15 m × 0.53 mm × 1.0 *μ*m; RESTEK, Bellefonte, USA) were used in this analysis. The IMS and column temperatures were 45 and 60°C, respectively. High-purity nitrogen (purity = 99.999%) was used as the carrier gas. A total of 3 g muscle sample was weighed accurately and placed in a vial (20 mL). The samples were then incubated at 60°C for 15 min (500 *r*/min). The automatic injection needle temperature was 85°C, and a final sample of 500 *μ*L gas was injected into the machine. A major software VOCal and three plug-in, namely, Reporter, Gallery Plot, and Dynamic PCA, were used to visualize the results.

### 2.8. Texture Profile Analysis (TPA) and Water-Holding Capacity (WHC) in the Muscle

The texture profile by Texture Analyser (TMS—PRO, Food Technology Corporation) was measured based on 3.0 × 2.0 cm^2^ sliced muscle samples. The measurement condition consisted of a 25N load cell, 8 mm cylinder probe, 30% deformation rate, and a double cycle at a constant rate of 30 mm/min. The instrument software output parameters including hardness, gumminess, springiness, cohesiveness, adhesiveness, and chewiness.

Six pieces of flesh (about 3 g) were sampled from the dorsal muscle to measure the water-holding capacity: The flesh sample (*W*1) was steamed for 5 min or centrifuged at 3,000 *r*/min for 10 min, then wiped off the surface liquid and weighed (*W*2) to calculate cooking loss and centrifugal loss. The cooking (centrifugal) loss (%) = 100 × (*W*1 − *W*2)/*W*1.

### 2.9. Statistical Analyses

All percentage data were arcsine transformed before analysis. The data were analyzed with one-way ANOVA followed by Tukey's test to analyze the differences among the treatments. Differences are determined as significant when *P* < 0.05. All data results are presented as means ± standard error.

## 3. Results

### 3.1. Growth Performances, Body Compositions, and Somatic Indices

No significant differences were observed in survival, feed efficiency, weight gain, specific growth rate, and somatic indices of fish from different groups (*P* > 0.05, Table 4). However, the weight gain in group MO-10PBM was slightly lower compared to the other groups.

The MO and PBM supplementation had mild effects on the proximate compositions of whole body, muscle, and liver (Table 5). Dietary MO supplementation significantly increased the moisture content of the liver (*P* < 0.05, Table 5).

### 3.2. Serum and Muscle Biochemical Parameters

In serum, no significant difference was observed in TG, TC, TBA, and PC of fish among different groups. However, the serum MDA content was significantly decreased by MO (*P* < 0.05, Table 6). There were no significant differences in muscle MDA and PC contents among dietary groups (*P* > 0.05, Table 6).

### 3.3. Mitochondrial DNA Copy Number

**Figure 1:**
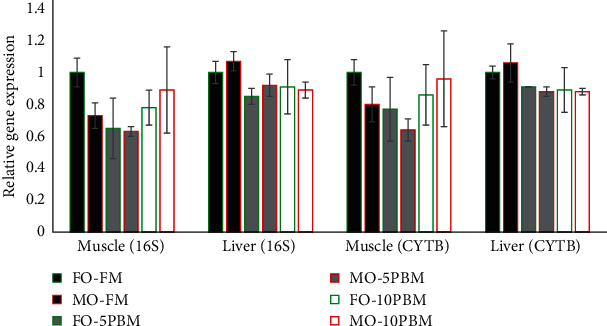
Mitochondrial DNA copy number (relative gene expression of 16S rRNA and cytochrome B in mitochondrial DNA) in the liver and muscle of experimental tiger puffer. Green frame: FO treatment; red frame: MO treatment; black fill: FM treatment; gray fill: 5% PBM treatment; white fill: 10% PBM treatment.

The MO and PBM supplementation did not significantly affect the relative gene expression of 16S rRNA and cytochrome B in the mitochondrial DHA of both muscle and liver (Figure 1).

### 3.4. Muscle Texture and Water-Holding Capacity

The MO and PBM supplementation did not significantly affect the hardness, adhesiveness, cohesiveness, springiness, gumminess, chewiness, cooking loss ratio, and centrifugal loss ratio of fish muscle (*P* > 0.05, Table 7).

### 3.5. Fatty Acid Composition in Muscle and Liver

In the muscle, dietary MO significantly (*P* < 0.05) increased the C18 : 2n-6 content, but significantly (*P* < 0.05) decreased the contents of C22 : 6n-3 (DHA; Table 8).

In the liver, dietary MO significantly (*P* < 0.05) increased the contents of C12 : 0, C14 : 0, C18 : 1n−9, and C18 : 2n-6, but significantly (*P* < 0.05) decreased the contents of C16 : 1n-7, C18 : 3n-3, ARA, C20 : 5n-3 (EPA), C22 : 5n-3, and DHA (Table 9). Dietary PBM significantly (*P* < 0.05) increased the C18 : 1n-9 content.

### 3.6. Free Amino Acids Composition in Muscle

Both dietary MO and PBM resulted in very few changes in amino acid composition of fish muscle (Table 10).

### 3.7. Volatile Flavor Components in the Muscle

**Figure 2:**
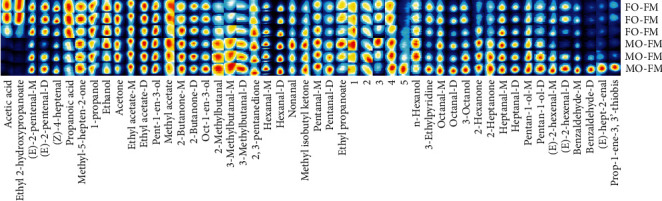
Gallery plot of volatile compounds in muscle of the FO-FM group and MO-FM group. The brightness indicates relative compound abundance. A column represents the signal peak of a certain volatile organic compound in different samples. A line represents all signal peaks of volatile organic compound selected from a certain sample. Compounds named as numbers were not successfully identified.

**Figure 3:**
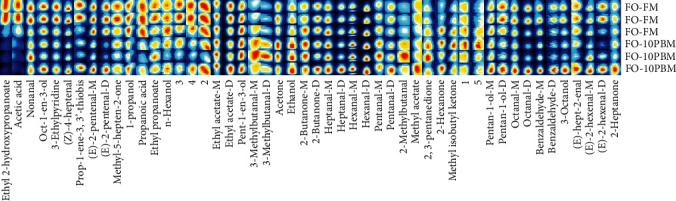
Gallery plot of volatile compounds in muscle of the FO-FM group and FO-10PBM group. The brightness indicates relative compound abundance. A column represents the signal peak of a certain volatile organic compound in different samples. A line represents all signal peaks of volatile organic compound selected from a certain sample. Compounds named as numbers were not successfully identified.

Three characteristic groups, namely, FO-FM, MO-FM, and FO-10PBM, were subjected to the analysis of muscle volatile flavor components, in order to determine the influences of MO and PBM. From all the muscle samples, 49 volatile flavor components were detected, of which 45 were identified successfully (Figures 2 and 3, Table S1). Compared to the FO-FM group, the MO-FM group had lower abundance of acetic acid, ethyl 2-hydroxypropanoate, (Z)-4-heptenal, and propanoic acid, but higher abundance of 2-methylbutanal, 3-methylbutanal dimer, 3-methylbutanal monomer, methyl isobutyl ketone, pentanal monomer, pentanal dimer, *n*-hexanol, octanal dimer, 2-hexanone, 2-heptanone, hexanal dimer, and pentan-1-ol dimer (Figure 2). Compared to the FO-FM group, the FO-10PBM group showed lower abundance of ethyl 2-hydroxypropanoate, acetic acid, (E)-2-pentenal dimer, (E)-2-pentenal monomer, methyl-5-hepten-2-one, 1-propanol, and propanoic acid, but higher abundance of 3-methylbutanal monomer, 3-methylbutanal dimer, 2-methylbutanal, and 2,3-pentanedione (Figure 3).

**Figure 4:**
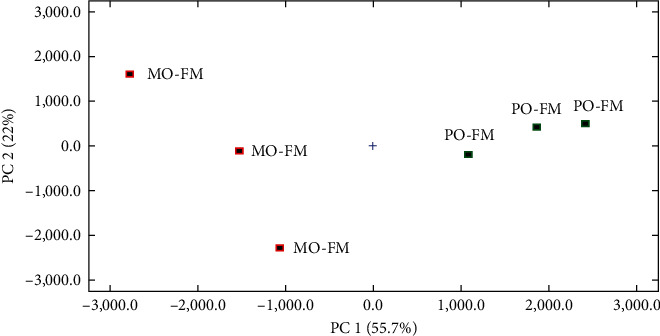
Principal component analysis (PCA) in volatile compounds in the muscle of the FO-FM group and MO-FM group.

**Figure 5:**
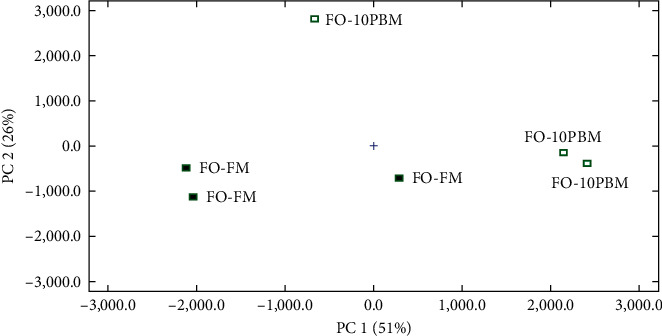
Principal component analysis (PCA) in volatile compounds in the muscle of the FO-FM group and FO−10PBM group.

The principal component analysis (PCA) showed that the muscle volatile flavor components clustered separately depending on dietary lipid source (Figure 4) or protein source (Figure 5).

## 4. Discussion

The current study clearly showed that 22% FM replacement (10% of diet) and 100% replacement of added oil (67% of total dietary lipid) with PBM and MO did not comprise the growth performance of farmed tiger puffer, indicating the great potential of this replacement strategy. Results of the present study were consistent with the results observed in other fish species. As mentioned above, PBM can replace 25%–70% of FM in fish feeds, while PO can replace FO at higher levels (usually 30%–100%). Low percentage replacement of dietary FM by PBM also had no significant effect on the growth of gilthead sea bream (*Sparus aurata*, <50% replacement) [52, 53], gibel carp (*Carassius auratus gibelio*, <50% replacement) [54], cuneate drum (*Nibea miichthioides*, <50% replacement) [55], and obscure pufferfish (<30% replacement) [11]. Complete FO replacement by PO had no significant effect on the growth of brown trout (*Salmo trutta*) [25], pacific white shrimp (*Litopenaeus vannamei*) [56], largemouth bass (*Micropterus salmoides*) [27], and barramundi (*Lates calcarifer*) [57].

Specific to tiger puffer, limited studies have shown that soybean meal and fish protein hydrolysate can replace a certain percentage (30% and 42.8%, respectively) of FM in tiger puffer diets [33, 50]. As for the application of alternative lipid sources in tiger puffer diets, our previous studies have shown that PO and soybean oil can replace 100% added FO in the diets of tiger puffer [50]. That was partly why PO was further tested in this study. However, 100% replacement of the 6% added FO with other alternative lipid sources such as linseed oil, rapeseed oil, and beef tallow significantly reduced the growth of juvenile tiger puffer [33]. The present study also indicates that combined use of PBM and PO was feasible. Similarly, for Atlantic salmon (*Salmo salar*), the replacement of 50% FO and FM in the diet by PO and PBM also had no significant effects on the growth performance [13]. Nevertheless, despite the high potential of the combined use of PBM and PO in fish diets, the present results still showed a decreasing trend (without significant differences) in growth with increasing PBM and PO levels. In a longer feeding period, significant growth reduction induced by PBM and PO may be observed.

The fish body composition was not obviously affected by dietary supplementation of both PBM and MO. However, it should be noted that dietary MO increased the moisture content in the liver. The increase of lipid content by dietary MO was consistent with a previous study on PO substitution for FO in tiger puffer [50]. The increase of liver moisture content could be related to the (although not significant) decrease of lipid content. However, this result was in contrast with other studies on alternative lipid sources which showed that FO replacement by alternative lipid sources easily causes an increase in lipid content in fish liver [30].

The fatty acid composition of experimental tiger puffer generally reflected those of the diets, as observed in the other studies [30, 35]. In this study, CCO was blended into PO to balance the composition of MUFA and SFA, considering that CCO is rich in SFA typically C12 : 0 [58]. The contents of C12 : 0 and C14 : 0 in the liver but not the muscle of tiger puffer were increased by dietary MO. The SFA content in tiger puffer muscle was not obviously affected by dietary MO, indicating that the excess SFA in MO may be readily utilized by fish. This provided evidence for the omega-3 fatty acids sparing effects of SFA, which have been widely observed in other fish studies [39, 40, 59–64]. Nevertheless, dietary MO still decreased the DHA and EPA contents in the muscle (EPA, by 15.6%; DHA, by 30.3%) and especially the liver (EPA, by 50.1%; DHA, by 50.5%). Attention should be paid on this if the fillet quality is considered. However, on the other hand, this may contribute to the lower malondialdehyde (MDA), which is a product of lipid peroxidation, content in the serum of the MO group. In other studies on tiger puffer, it was observed that the FO replacement with rapeseed oil also reduced the MDA level [33]. Since, LC-PUFA are more susceptible to peroxidation compared to SFA and MUFA, the fish oil, which is rich in LC-PUFA, is under higher peroxidation pressure. The lower MDA content in the muscle of fish fed alternative oils is a favorable quality trait. This advantage could be more significant in longer term experiments considering that the MDA accumulates in fish muscle.

Free amino acid (FAA) is an important flavor component in fish flesh products. When the FO was replaced by MO in the diet of tiger puffer, the lysine content in muscle tended to increase and the histidine and threonine contents tended to decrease. Lysine is a sweet amino acid and histidine is a bitter amino acid [65]. Therefore, it was speculated that the sweetness can be increased but the bitterness can be reduced by dietary MO. In all groups, taurine was the most abundant FAA. Similar results were observed in sea bass (*Dicentrarchus labrax*) [66] and gibel carp [67]. However, it seemed that taurine has no effect on the taste or the formation of aromatic active ingredients [66].

Besides FFA, volatile organic compounds also have great influence on fish flesh quality. The identified volatile flavor compounds in tiger puffer mainly consist of aldehyde, alcohol, ketone, and ester compounds. The volatile flavor compound composition was clearly changed by both MO and PBM. The MO group had lower abundance of (Z)-4-heptenal, but higher abundance of 2-methylbutanal, 3-methylbutanal dimer, 3-methylbutanal monomer, pentanal monomer, pentanal dimer, *n*-hexanol, octanal dimer, and hexanal dimer. (Z)-4-heptenal is derived from the lipid oxidation of n-3 PUFA [68], which usually indicates the deterioration of fish and presents flavor of boiled fish and fatty grease. The lower levels of (Z)-4-heptanal in the MO and PBM groups may be due to the fact that the control group contained a higher proportion of n-3 PUFA that was more easily oxidized. Therefore, the addition of MO or PBM to the diet will reduce the adverse flavors caused by lipid oxidation. The 2-methylbutanal, which has strong burnt flavor, may be related to the degradation of amino acid [69]. The 3-methylbutanal, which was also higher in abundance in the MO group, has green grass, vegetables, almond, and malt flavors. Pentanal, which is probably derived from n-6 PUFA oxidation [70], has a pungent flavor. Both octanal and hexanal have grassy, leafy, fruity, and other plant flavors [71]. The PBM group had lower abundance of 1-octene-3-ol, nonanal, (E)-2-pentenal dimer, and (E)-2-pentenal monomer, but higher abundance of 3-methylbutanal monomer, 3-methylbutanal dimer, 2-methylbutanal, and 2,3-pentanedione. The 1-octene-3-ol, which may result in the flavors of fishy, fatty, and mushroom, is a product of oxidation of linoleic acid or other polyunsaturated fatty acid [68, 69, 72]. Nonanal, showing geranium, plastic, and marine flavors, is the product of oxidation of oleic acid and linoleic acid [73]. The above results showed that the effects of FM and FO replacement by PBM and MO resulted in both pleasant and unpleasant changes in flavor. The overall influence on fish flesh flavor needs to be comprehensively evaluated by other parameters, in particular by a sensory evaluation.

In conclusion, combined replacement of FM and FO by PBM and MO had no significant effect on the growth performance and body proximate composition of tiger puffer. The supplementation of both PBM and MO significantly decreased the malondialdehyde content in serum. The FM and FO replacement by PBM and MO also reduced the fillet volatile flavor compounds derived from PUFA oxidation, such as (Z)-4-heptenal, 1-octene-3-ol, and nonanal. Further studies examining higher FM replacement levels by PBM are recommended.

## Figures and Tables

**Table 1:** Formulation and proximate composition of the experimental diets (% dry matter basis).

Ingredients	FO-FM	MO-FM	FO-5PBM	MO-5PBM	FO-10PBM	MO-10PBM
Fish meal^1^	45	45	40	40	35	35
Poultry byproduct meal^1^	0	0	5	5	10	10
Corn gluten meal^2^	7	7	7	7	7	7
Soybean meal^3^	7	7	7	7	7	7
Dephenolized cottonseed protein^4^	7	7	7	7	7	7
Wheat meal^5^	19.68	19.68	19.68	19.68	19.68	19.68
Brewer's yeast^6^	5	5	5	5	5	5
Mineral premix^7^	0.5	0.5	0.5	0.5	0.5	0.5
Vitamin premix^7^	1	1	1	1	1	1
Monocalcium phosphate^7^	1	1	1	1	1	1
L-ascorbyl-2-polyphosphate^7^	0.2	0.2	0.2	0.2	0.2	0.2
Choline chloride^7^	0.2	0.2	0.2	0.2	0.2	0.2
Attractant^7^	0.3	0.3	0.3	0.3	0.3	0.3
Ethoxyquin^7^	0.02	0.02	0.02	0.02	0.02	0.02
Mold inhibitor^7^	0.1	0.1	0.1	0.1	0.1	0.1
Soya lecithin^7^	1	1	1	1	1	1
Fish oil	5	0	5	0	5	0
Mixed oil^8^	0	5	0	5	0	5
Proximate composition						
Crude protein	48.00	47.90	47.02	48.34	47.89	47.92
Crude lipid	7.24	7.48	7.88	7.52	7.51	8.14
Ash	10.34	10.43	10.01	10.24	9.81	9.92

^1^The Pollock meal had a protein content of 69.0% and a lipid content of 9.9% (of dry matter). The chicken byproduct meal had a protein content of 66.5% and a lipid content of 13.9% (of dry matter). ^2^The corn gluten meal had protein content of 65.4% and a lipid content of 0.7% (of dry matter). ^3^The soybean meal had protein content of 52.2% and a lipid content of 1.7% (of dry matter). ^4^The dephenolized cottonseed protein had protein content of 64.2% and a lipid content of 10.9% (of dry matter). ^5^The wheat meal had protein content of 15.1% and a lipid content of 1.1% (of dry matter). ^6^The Brewer's yeast had protein content of 53.7% and a lipid content of 2.2% (of dry matter). ^7^Vitamin premix, mineral premix, and other additives were purchased from Qingdao Surgreen Bioengineering Co. Ltd. ^8^Mixed oil: 1 : 1 mixture of poultry oil and coconut oil. The oils were purchased from the Shandong Haiding Agriculture and Animal Husbandry Co., Ltd. FO, fish oil; FM, fishmeal; MO, mixed oil; PBM, poultry byproduct meal.

**Table 2:** Fatty acids composition of fish oil, mixed oil, and experimental diets (% total fatty acid).

Fatty acid	FO	MO	FO-FM	MO-FM	FO-5PBM	MO-5PBM	FO-10PBM	MO-10PBM
12 : 0	0.06	26.2	0.08	11.6	0.08	11.5	0.09	10.8
14 : 0	5.18	9.92	4.62	6.61	4.35	6.10	4.23	5.62
16 : 0	18.2	17.9	21.9	19.2	22.5	20.3	24.8	22.1
18 : 0	4.36	5.57	5.06	4.95	5.32	5.41	5.90	5.94
∑SFA	29.4	59.6	32.3	42.3	32.9	43.5	35.7	44.6
16 : 1n-7	5.31	2.67	4.96	3.27	5.02	3.49	5.28	3.93
18 : 1n-9	14.7	26.1	14.7	17.7	17.0	20.7	19.5	21.8
20 : 1n-9	3.98	0.39	1.79	1.08	1.68	1.18	1.71	1.27
∑MUFA	29.4	29.4	21.5	22.0	23.7	25.3	26.5	27.1
18 : 2n-6	11.7	10.5	11.8	14.6	12.3	16.2	14.5	17.1
20 : 2n-6	0.37	ND	0.33	0.21	0.33	0.43	0.29	0.39
20 : 3n-6	5.26	ND	0.08	1.39	0.10	0.09	0.11	0.09
20 : 4n-6	0.13	0.15	0.91	1.24	0.89	1.60	0.91	0.95
∑n-6PUFA	21.2	10.6	13.1	17.5	13.6	18.4	15.8	18.5
18 : 3n-3	1.97	0.20	2.79	0.49	2.72	0.54	2.60	0.74
20 : 5n-3	7.33	ND	9.53	3.71	7.26	3.35	5.86	3.08
22 : 5n-3	1.47	ND	4.32	0.77	4.94	0.67	1.36	0.66
22 : 6n-3	13.8	ND	13.0	9.89	11.9	4.41	8.87	4.27
∑n-3PUFA	25.6	0.20	29.7	14.9	26.8	8.97	18.7	8.75
∑n-3/∑n-6	1.21	0.02	2.26	0.85	1.96	0.49	1.18	0.47

FO, fish oil; FM, fishmeal; MO, mixed oil; PBM, poultry byproduct meal; SFA, saturated fatty acid; MUFA, mono-unsaturated fatty acid; PUFA, poly-unsaturated fatty acid; ND, nondetectable.

**Table 3:** Sequences information of the primers used in this work.

Primer	Sequence (5′−3′)	GenBank reference	PL (bp)
16S rRNA-F	ATGTGGACCTGTATGAATGGC	NC 004299.1	119
16S rRNA-R	CTCCATAGGGTCTTCTCGTCTT		

CYTB-F	CCTCCTGGGCTTCACAATCA	NC 004299.1	123
CYTB-R	TTAATGTGGGCGGGGGTAAC		

*β*-Actin-F	GACGCAAAACCTCCGAACTG	Gene ID 101,079,312	129
*β*-Actin-R	CCTCCAAACGGATCAGCACA		

EF1*α*-F	TGGCCTTTAGCCGAATGAGG	Gene ID 653,026	117
EF1*α*-R	TGTCGGGCCAATCAATCCAG		

PL, product length; CYTB, cytochrome B.

**Table 4:** Growth performance and somatic parameters of experimental tiger puffer (mean ± standard error).

Parameter	FO-FM	MO-FM	FO-5PBM	MO-5PBM	FO-10PBM	MO-10PBM	*P*-value
IBW (g)	14.2 ± 0.18	14.2 ± 0.10	14.4 ± 0.13	14.2 ± 0.03	14.5 ± 0.04	14.4 ± 0.03	0.256
FBW (g)	76.3 ± 1.36	72.2 ± 2.06	73.7 ± 4.65	73.0 ± 0.41	72.5 ± 2.05	68.0 ± 4.02	0.533
Survival (%)	98.3 ± 1.67	99.0 ± 1.11	100 ± 0.00	100 ± 0.00	100 ± 0.00	98.3 ± 1.67	0.743
WG (%)	437 ± 2.99	410 ± 13.8	412 ± 27.6	413 ± 1.65	400 ± 15.6	372 ± 27.1	0.343
SGR (%/d)	3.73 ± 0.01	3.62 ± 0.06	3.63 ± 0.12	3.63 ± 0.01	3.57 ± 0.07	3.44 ± 0.13	0.341
FI (%/d)	2.61 ± 0.04	2.54 ± 0.02	2.50 ± 0.01	2.56 ± 0.02	2.54 ± 0.04	2.46 ± 0.04	0.111
FCR	0.85 ± 0.03	0.83 ± 0.03	0.82 ± 0.02	0.84 ± 0.00	0.84 ± 0.04	0.81 ± 0.04	0.755
HSI (%)	10.7 ± 0.42	10.4 ± 0.48	11.3 ± 0.95	10.4 ± 0.76	10.4 ± 0.82	10.0 ± 0.72	0.841
VSI (%)	15.7 ± 0.30	15.6 ± 0.41	16.9 ± 0.98	15.2 ± 1.17	15.2 ± 1.84	15.2 ± 0.98	0.822
CF (g/cm^3^)	3.63 ± 0.06	3.63 ± 0.12	3.83 ± 0.03	3.49 ± 0.13	3.62 ± 0.10	3.50 ± 0.06	0.322

IBW, initial body weight; FBW, final body weight; WG, weight gain; SGR, specific growth rate; FI, feed intake; FCR, feed conversion ratio; HSI, hepatosomatic index; VSI, viscerosomatic index; CF, condition factor; WG = (final weight − initial weight)/initial weight × 100; SGR = (ln(final weight) − ln(initial weight))/days of experiment × 100; FI = total dry feed intake/(days of experiment × (initial weight + final weight)/2) × 100; FCR = (final weight − initial weight)/total feed intake; CF = body weight/body length^3^ × 100; HSI = liver weight/body weight × 100; VSI = visceral weight/body weight × 100.

**Table 5:** Proximate composition of whole fish, muscle and liver in experimental tiger puffer (% wet weight, mean ± standard error).

Parameter	FO-FM	MO-FM	FO-5PBM	MO-5PBM	FO-10PBM	MO-10PBM	*P*-value
Whole fish							
Moisture	74.7 ± 0.23	75.1 ± 0.30	74.7 ± 0.85	75.0 ± 0.22	75.0 ± 0.08	75.3 ± 0.19	0.905
Crude lipid	5.25 ± 0.25	5.58 ± 0.11	6.63 ± 0.64	5.64 ± 0.13	5.68 ± 0.20	5.28 ± 0.37	0.122
Crude protein	15.7 ± 0.51	16.7 ± 0.21	16.1 ± 0.46	15.8 ± 0.40	15.7 ± 0.24	16.2 ± 0.27	0.454
Ash	2.39 ± 0.05	2.46 ± 0.02	2.32 ± 0.04	2.36 ± 0.08	2.40 ± 0.05	2.41 ± 0.01	0.728
Muscle							
Moisture	79.9 ± 0.35	78.1 ± 0.16	79.9 ± 0.04	79.8 ± 0.30	80.0 ± 0.66	78.4 ± 0.13	0.960
Crude lipid	0.46 ± 0.05	0.45 ± 0.01	0.46 ± 0.01	0.51 ± 0.05	0.53 ± 0.02	0.51 ± 0.02	0.317
Crude protein	17.7 ± 0.56	19.3 ± 0.10	17.7 ± 0.06	17.7 ± 0.25	17.6 ± 0.74	19.0 ± 0.22	0.064
Liver							
Moisture	26.8 ± 1.20^a^	32.0 ± 0.60^b^	29.7 ± 0.53^ab^	31.1 ± 1.21^ab^	28.2 ± 0.68^ab^	30.7 ± 1.18^ab^	0.031
Crude lipid	57.4 ± 1.83	57.3 ± 1.95	61.5 ± 3.74	55.1 ± 2.52	60.7 ± 2.31	54.5 ± 1.63	0.335

Data in a same row not sharing a same superscript letter were significantly different (one-way ANOVA).

**Table 6:** Serum and muscle biochemical indices of experimental tiger puffer (mean ± standard error).

Parameter	FO-FM	MO-FM	FO-5PBM	MO-5PBM	FO-10PBM	MO-10PBM	*P*-value
Serum							
TG (mmol/L)	1.15 ± 0.07	0.74 ± 0.09	0.86 ± 0.00	1.05 ± 0.28	0.89 ± 0.31	0.52 ± 0.02	0.232
TC (mmol/L)	9.13 ± 0.32	8.24 ± 0.23	8.04 ± 0.51	7.52 ± 1.44	7.72 ± 0.66	6.25 ± 0.06	0.179
TBA (*µ*mol/L)	5.60 ± 0.40	5.29 ± 0.51	5.84 ± 0.21	6.14 ± 0.60	6.31 ± 0.63	5.03 ± 0.34	0.519
MDA (nmol/ml)	6.48 ± 0.33^b^	4.23 ± 0.12^a^	5.45 ± 0.19^b^	3.96 ± 0.23^a^	5.66 ± 0.06^b^	3.76 ± 0.34^a^	<0.001
PC (nmol/mg)	0.27 ± 0.02	0.38 ± 0.15	0.47 ± 0.01	0.38 ± 0.08	0.32 ± 0.12	0.25 ± 0.05	0.518
Muscle							
MDA (nmol/g)	0.95 ± 0.22	0.93 ± 0.18	0.91 ± 0.29	0.83 ± 0.10	0.99 ± 0.11	1.10 ± 0.11	0.923
PC (nmol/mg)	0.72 ± 0.01	0.66 ± 0.07	0.61 ± 0.02	0.66 ± 0.05	0.60 ± 0.02	0.51 ± 0.02	0.079

TG, triacylglycerol; TC, total cholesterol; HDL-C, high-density lipoprotein cholesterol; LDL-C, low-density lipoprotein cholesterol; TBA, total bile acid; MDA, malondialdehyde; PC, protein carbonyl. ^a,b^Data in a same row not sharing a same superscript letter were significantly different (one-way ANOVA).

**Table 7:** Muscle texture and water-holding capacity of experimental tiger puffer (mean ± standard error).

Parameter	FO-FM	MO-FM	FO-5PBM	MO-5PBM	FO-10PBM	MO-10PBM	*P*-value
Hardness (N)	4.25 ± 0.38	3.21 ± 0.41	5.15 ± 1.26	4.12 ± 0.11	4.12 ± 0.02	3.70 ± 1.15	0.486
Adhesiveness (mJ)	0.04 ± 0.00	0.05 ± 0.00	0.04 ± 0.00	0.04 ± 0.00	0.04 ± 0.00	0.05 ± 0.00	0.051
Cohesiveness (Ratio)	0.41 ± 0.01	0.41 ± 0.00	0.41 ± 0.02	0.45 ± 0.01	0.42 ± 0.00	0.40 ± 0.01	0.995
Springiness (mm)	1.30 ± 0.11	1.15 ± 0.06	1.32 ± 0.17	1.47 ± 0.00	1.41 ± 0.27	1.10 ± 0.07	0.943
Gumminess (N)	1.78 ± 0.18	1.31 ± 0.16	2.15 ± 0.62	2.05 ± 0.13	1.75 ± 0.01	1.51 ± 0.51	0.691
Chewiness (mJ)	2.41 ± 0.44	1.54 ± 0.20	2.95 ± 1.18	2.71 ± 0.12	2.47 ± 0.48	1.71 ± 0.66	0.704
Centrifugal water loss (%)	14.0 ± 2.31	13.4 ± 0.22	15.2 ± 0.95	14.2 ± 1.64	16.4 ± 1.08	13.1 ± 0.75	0.625
Cooking loss (%)	34.2 ± 0.94	35.8 ± 0.47	37.6 ± 1.93	36.9 ± 0.41	37.0 ± 1.06	34.6 ± 0.16	0.137

**Table 8:** Fatty acid compositions in the muscle of experimental tiger puffer (% total fatty acids, mean ± standard error).

Fatty acid	FO-FM	MO-FM	FO-5PBM	MO-5PBM	FO-10PBM	MO-10PBM	*P*-value
C12 : 0	0.00 ± 0.00	0.00 ± 0.00	0.00 ± 0.00	0.24 ± 0.24	0.18 ± 0.18	0.00 ± 0.00	0.679
C14 : 0	1.76 ± 0.80	1.68 ± 0.21	0.71 ± 0.06	1.60 ± 0.04	0.35 ± 0.18	1.44 ± 0.04	0.133
C16 : 0	21.6 ± 0.90	22.3 ± 0.31	22.8 ± 0.23	21.6 ± 0.70	21.7 ± 0.22	21.8 ± 0.86	0.763
C18 : 0	9.78 ± 0.83	11.3 ± 0.09	10.7 ± 0.22	11.1 ± 0.21	10.6 ± 0.26	11.3 ± 0.13	0.173
∑SFA	40.5 ± 1.73	38.8 ± 0.64	38.1 ± 0.07	38.5 ± 0.39	38.0 ± 0.83	39.2 ± 0.47	0.486
C16 : 1n-7	0.80 ± 0.40	0.78 ± 0.39	1.19 ± 0.04	1.04 ± 0.12	0.71 ± 0.35	0.56 ± 0.56	0.874
C18 : 1n-9	13.6 ± 1.89	14.4 ± 0.15	12.7 ± 0.07	14.9 ± 0.72	14.2 ± 0.18	16.1 ± 0.50	0.412
∑MUFA	14.4 ± 1.49	15.2 ± 0.39	13.9 ± 0.11	15.9 ± 0.84	14.9 ± 0.26	16.7 ± 1.06	0.429
C18 : 2n-6	8.48 ± 0.70^a^	17.2 ± 0.79^b^	10.6 ± 0.41^a^	17.2 ± 1.67^b^	11.2 ± 0.30^a^	18.7 ± 1.06^b^	<0.001
C20 : 4n-6	1.23 ± 0.62	1.05 ± 0.53	2.27 ± 0.06	1.55 ± 0.08	2.46 ± 0.03	2.11 ± 0.24	0.127
∑*n*-6PUFA	9.72 ± 1.30^a^	18.2 ± 1.32^bc^	12.8 ± 0.47^ab^	18.8 ± 1.74^bc^	13.7 ± 0.27^ab^	20.8 ± 0.82^c^	0.001
C20 : 5n-3	5.17 ± 0.88	5.11 ± 0.26	6.13 ± 0.14	4.73 ± 0.00	5.64 ± 0.15	4.13 ± 0.11	0.162
C22 : 5n-3	3.73 ± 0.26	3.91 ± 0.13	3.92 ± 0.00	3.87 ± 0.20	3.77 ± 0.08	3.48 ± 0.50	0.795
C22 : 6n-3	23.3 ± 0.83^c^	16.5 ± 1.50^abc^	21.8 ± 1.12^bc^	15.6 ± 1.92^ab^	21.1 ± 0.74^bc^	13.6 ± 1.99^a^	0.004
∑n-3PUFA	32.2 ± 1.92^c^	25.5 ± 1.12^abc^	31.8 ± 0.98^c^	24.2 ± 1.72^ab^	30.5 ± 0.84^bc^	21.2 ± 1.38^a^	0.002
∑n-3/∑n-6	3.39 ± 0.30^d^	1.42 ± 0.18^ab^	2.48 ± 0.17^cd^	1.33 ± 0.20^ab^	2.23 ± 0.09^bc^	1.02 ± 0.11^a^	<0.001

Data in a same row not sharing a same superscript letter were significantly different (one-way ANOVA).

**Table 9:** Fatty acid compositions in the liver of experimental tiger puffer (% total fatty acids, mean ± standard error).

Fatty acid	FO-FM	MO-FM	FO-5PBM	MO-5PBM	FO-10PBM	MO-10PBM	*P*-value
C12 : 0	0.01 ± 0.01^a^	2.87 ± 0.16^b^	0.00 ± 0.00^a^	2.77 ± 0.22^b^	0.00 ± 0.00^a^	2.72 ± 0.01^b^	<0.001
C14 : 0	3.02 ± 0.17^a^	5.98 ± 0.19^b^	2.74 ± 0.05^a^	5.57 ± 0.18^b^	2.88 ± 0.06^a^	5.53 ± 0.05^b^	<0.001
C16 : 0	23.2 ± 1.09	24.0 ± 1.34	24.3 ± 1.56	24.6 ± 0.77	24.0 ± 0.39	24.5 ± 0.01	0.916
C18 : 0	11.0 ± 0.18^b^	11.3 ± 0.21^b^	10.8 ± 0.14^b^	11.0 ± 0.45^b^	9.33 ± 0.15^a^	10.2 ± 0.20^ab^	0.003
∑SFA	37.7 ± 1.05^ab^	44.4 ± 1.58^c^	38.3 ± 1.48^ab^	44.1 ± 1.01^c^	36.5 ± 0.61^a^	43.1 ± 0.24^bc^	0.001
C16 : 1n-7	6.07 ± 0.23^ab^	5.27 ± 0.14^a^	6.06 ± 0.05^ab^	5.51 ± 0.37^a^	6.74 ± 0.16^b^	5.45 ± 0.10^a^	0.008
C18 : 1n-9	19.8 ± 0.55^a^	23.1 ± 0.05^cd^	20.4 ± 0.01^ab^	24.6 ± 0.54^de^	22.0 ± 0.16^bc^	25.6 ± 0.08^e^	<0.001
C20 : 1n-9	1.21 ± 0.04	1.12 ± 0.05	1.18 ± 0.13	1.05 ± 0.07	1.28 ± 0.07	1.11 ± 0.03	0.230
∑MUFA	27.1 ± 0.72^a^	29.5 ± 0.22^bc^	27.7 ± 0.19^ab^	31.2 ± 0.25^cd^	30.0 ± 0.06^c^	32.2 ± 0.22^d^	<0.001
C18 : 2n-6	9.97 ± 0.23^a^	12.3 ± 0.35^bc^	10.0 ± 0.46^a^	12.6 ± 0.27^c^	11.1 ± 0.21^ab^	13.1 ± 0.17^c^	<0.001
C20 : 2n-6	0.59 ± 0.06	0.63 ± 0.04	0.57 ± 0.02	0.61 ± 0.05	0.60 ± 0.02	0.66 ± 0.07	0.887
C20 : 4n−6	0.46 ± 0.02^b^	0.28 ± 0.03^a^	0.49 ± 0.04^b^	0.28 ± 0.02^a^	0.48 ± 0.02^b^	0.27 ± 0.00^a^	<0.001
∑*n*-6PUFA	11.0 ± 0.31^a^	13.2 ± 0.42^bc^	11.1 ± 0.52^a^	13.5 ± 0.31^bc^	12.2 ± 0.21^ab^	14.1 ± 0.24^c^	<0.001
C18 : 3n-3	2.11 ± 0.03^b^	0.97 ± 0.05^a^	2.01 ± 0.02^b^	0.98 ± 0.15^a^	1.94 ± 0.02^b^	1.00 ± 0.06^a^	<0.001
C20 : 5n-3	4.77 ± 0.38^b^	2.44 ± 0.2^a^	4.3 ± 0.43^b^	2.11 ± 0.11^a^	3.92 ± 0.08^b^	1.84 ± 0.02^a^	<0.001
C22 : 5n-3	3.34 ± 0.32^abc^	2.24 ± 0.22^ab^	3.69 ± 0.35^bc^	2.20 ± 0.15^a^	3.71 ± 0.41^c^	1.91 ± 0.08^a^	0.004
C22 : 6n-3	11.2 ± 0.87^b^	5.71 ± 0.50^a^	10.5 ± 0.65^b^	4.96 ± 0.31^a^	9.38 ± 0.27^b^	4.38 ± 0.24^a^	<0.001
∑n-3PUFA	21.4 ± 1.38^b^	11.4 ± 0.96^a^	20.4 ± 0.76^b^	10.3 ± 0.70^a^	19.0 ± 0.71^b^	9.12 ± 0.13^a^	<0.001
∑n-3/∑n-6	1.95 ± 0.12^c^	0.86 ± 0.05^a^	1.85 ± 0.02^bc^	0.76 ± 0.05^a^	1.56 ± 0.04^b^	0.65 ± 0.02^a^	<0.001

Data in a same row not sharing a same superscript letter were significantly different (one-way ANOVA).

**Table 10:** Free amino acid and taurine compositions in the muscle of experimental tiger puffer (g/kg, dry matter basis, mean ± standard error).

Amino acid	FO-FM	MO-FM	FO-5PBM	MO-5PBM	FO-10PBM	MO-10PBM	*P*-value
Thr	1.37 ± 0.15	0.93 ± 0.04	1.10 ± 0.07	1.06 ± 0.03	1.09 ± 0.01	0.90 ± 0.11	0.094
Val	0.41 ± 0.08	0.25 ± 0.02	0.32 ± 0.05	0.29 ± 0.05	0.33 ± 0.00	0.29 ± 0.04	0.579
Met	0.75 ± 0.07	0.72 ± 0.08	0.78 ± 0.06	0.73 ± 0.02	0.68 ± 0.01	0.77 ± 0.02	0.961
Ile	0.13 ± 0.06	0.09 ± 0.03	0.11 ± 0.05	0.14 ± 0.02	0.15 ± 0.00	0.12 ± 0.05	0.539
Leu	0.20 ± 0.07	0.13 ± 0.01	0.17 ± 0.04	0.19 ± 0.01	0.20 ± 0.00	0.16 ± 0.04	0.490
Phe	0.86 ± 0.05	0.78 ± 0.06	0.77 ± 0.10	0.74 ± 0.01	0.71 ± 0.01	0.77 ± 0.03	0.265
Lys	3.99 ± 0.53	4.34 ± 0.12	3.08 ± 0.01	4.63 ± 0.15	3.80 ± 0.02	4.02 ± 0.04	0.506
His	0.28 ± 0.03^b^	0.16 ± 0.01^ab^	0.20 ± 0.00^ab^	0.21 ± 0.02^ab^	0.22 ± 0.01^ab^	0.14 ± 0.03^a^	0.025
Arg	0.94 ± 0.21	1.05 ± 0.04	0.91 ± 0.04	1.25 ± 0.14	1.27 ± 0.02	1.26 ± 0.09	0.192
∑EAA	8.93 ± 0.64	8.45 ± 0.21	7.43 ± 0.38	9.24 ± 0.19	8.46 ± 0.03	8.52 ± 0.20	0.399
Tau	34.63 ± 1.09	35.35 ± 1.82	33.96 ± 3.15	33.95 ± 2.76	33.37 ± 2.74	34.23 ± 0.04	0.968
Asp	0.30 ± 0.01	0.28 ± 0.02	0.32 ± 0.02	0.27 ± 0.01	0.29 ± 0.01	0.34 ± 0.00	0.551
Ser	0.22 ± 0.01	0.26 ± 0.03	0.24 ± 0.03	0.19 ± 0.04	0.23 ± 0.01	0.25 ± 0.03	0.507
Glu	0.38 ± 0.04	0.33 ± 0.03	0.41 ± 0.03	0.39 ± 0.06	0.42 ± 0.01	0.37 ± 0.02	0.782
Gly	1.98 ± 0.06^b^	2.11 ± 0.05^b^	1.93 ± 0.13^b^	1.58 ± 0.02^a^	2.47 ± 0.03^c^	2.75 ± 0.01^c^	0.041
Ala	1.87 ± 0.09^abc^	2.04 ± 0.04^bc^	1.96 ± 0.01^bc^	1.61 ± 0.25^ab^	1.37 ± 0.01^a^	2.15 ± 0.06^c^	0.788
Tyr	0.16 ± 0.05	0.11 ± 0.00	0.14 ± 0.04	0.15 ± 0.00	0.16 ± 0.01	0.14 ± 0.02	0.562
∑NEAA	39.5 ± 1.16	40.5 ± 1.84	34.0 ± 3.39	38.1 ± 2.38	38.0 ± 2.53	40.2 ± 0.12	0.928
∑AA	48.5 ± 0.52	48.9 ± 1.93	46.4 ± 3.77	47.4 ± 2.19	46.7 ± 2.75	48.7 ± 0.22	0.818

Data in a same row not sharing a same superscript letter were significantly different.

## Data Availability

Raw data supporting the conclusions of this manuscript will be made available by the authors, without undue reservation, to any qualified researcher.

## Supplementary Materials

Supplementary Materials Table S1: the relatively quantitative results of muscle volatile organic compounds.
