# Supplementary material for: Combined Replacement of Fishmeal and Fish Oil by Poultry Byproduct Meal and Mixed Oil: Effects on the Growth Performance, Body Composition, and Muscle Quality of Tiger Puffer
Source: Aquac Nutr. 2024 Feb 15;2024:1402602. doi: 10.1155/2024/1402602 (PMC10883743; doi:10.1155/2024/1402602)
Supplement: Supplementary Materials — Table S1: the relatively quantitative results of muscle volatile organic compounds. [file 1402602.f1.docx]

**Supplementary Table S1** The relatively quantitative results of muscle volatile organic compounds

| **Compound** | **CAS#** | **Formula** | **MW** | **RI** | **Rt [sec]** | **Comment** | **Peak Area** | | | | | | | | |
| --- | --- | --- | --- | --- | --- | --- | --- | --- | --- | --- | --- | --- | --- | --- | --- |
|  |  |  |  |  |  |  | **FO-FM** | **FO-FM** | **FO-FM** | **FO-10PBM** | **FO-10PBM** | **FO-10PBM** | **MO-FM** | **MO-FM** | **MO-FM** |
| Nonanal | C1241964 | C9H18O | 142.2 | 1109.1 | 514.44 |  | 849.74 | 860.73 | 665.37 | 707.51 | 831.34 | 760.48 | 1108.02 | 905.48 | 826.22 |
| Octanal-M | C124130 | C8H16O | 128.2 | 1009.9 | 365.25 | Monomer | 1213.70 | 1226.86 | 1045.65 | 975.21 | 1008.91 | 1348.80 | 1319.30 | 1260.53 | 1488.70 |
| Octanal-D | C124130 | C8H16O | 128.2 | 1007.7 | 362.43 | Dimer | 239.88 | 258.64 | 175.71 | 149.08 | 161.17 | 284.32 | 269.39 | 245.76 | 360.29 |
| 3-Octanol | C589980 | C8H18O | 130.2 | 988.7 | 341.25 |  | 757.79 | 760.99 | 608.35 | 607.66 | 543.74 | 914.60 | 678.12 | 939.89 | 1054.96 |
| oct-1-en-3-ol | C3391864 | C8H16O | 128.2 | 985.2 | 337.96 |  | 304.45 | 310.94 | 259.03 | 243.55 | 227.91 | 297.42 | 235.55 | 279.81 | 342.37 |
| 3-ethylpyridine | C536787 | C7H9N | 107.2 | 978.6 | 331.84 |  | 201.09 | 202.86 | 169.58 | 151.22 | 149.29 | 186.00 | 135.11 | 182.91 | 258.92 |
| Benzaldehyde-M | C100527 | C7H6O | 106.1 | 961.9 | 316.78 | Monomer | 583.51 | 589.16 | 445.31 | 358.58 | 351.65 | 470.76 | 365.08 | 484.59 | 678.23 |
| (E)-hept-2-enal | C18829555 | C7H12O | 112.2 | 949.9 | 306.43 |  | 90.51 | 98.59 | 91.51 | 76.35 | 62.57 | 105.33 | 86.79 | 117.75 | 229.08 |
| Heptanal-M | C111717 | C7H14O | 114.2 | 902.6 | 268.78 | Monomer | 1266.77 | 1278.70 | 1363.02 | 1337.99 | 1277.51 | 1556.71 | 1469.73 | 1504.26 | 1578.08 |
| Heptanal-D | C111717 | C7H14O | 114.2 | 900.7 | 267.37 | Dimer | 619.01 | 611.23 | 566.59 | 506.87 | 467.57 | 854.02 | 692.39 | 759.14 | 907.18 |
| ( Z)-4-heptenal | C6728310 | C7H12O | 112.2 | 898.2 | 265.48 |  | 1081.15 | 1069.85 | 772.11 | 680.04 | 656.24 | 892.29 | 633.31 | 774.96 | 787.51 |
| prop-1-ene-3,3'-thiobis | C592881 | C6H10S | 114.2 | 873.5 | 249.95 |  | 71.29 | 71.76 | 48.68 | 55.10 | 46.97 | 75.20 | 50.05 | 63.55 | 152.67 |
| ( E)-2-hexenal-M | C6728263 | C6H10O | 98.1 | 848.7 | 235.84 | Monomer | 223.07 | 231.03 | 213.47 | 193.00 | 263.79 | 317.98 | 193.01 | 297.62 | 359.08 |
| Hexanal-M | C66251 | C6H12O | 100.2 | 794.3 | 207.60 | Monomer | 2000.35 | 1963.50 | 2212.19 | 2357.77 | 2347.97 | 2479.98 | 2413.66 | 2428.17 | 2534.59 |
| Hexanal-D | C66251 | C6H12O | 100.2 | 794.3 | 207.60 | Dimer | 4714.93 | 4710.21 | 4720.31 | 4913.23 | 4768.39 | 5764.78 | 5781.07 | 5998.92 | 6162.31 |
| pentan-1-ol-M | C71410 | C5H12O | 88.1 | 760.3 | 191.49 | Monomer | 304.29 | 307.94 | 302.57 | 285.21 | 291.68 | 347.30 | 351.25 | 387.16 | 417.31 |
| (E)-2-pentenal-M | C1576870 | C5H8O | 84.1 | 751.6 | 187.52 | Monomer | 388.56 | 420.98 | 380.77 | 288.48 | 283.86 | 399.25 | 295.97 | 390.48 | 402.79 |
| (E)-2-pentenal-D | C1576870 | C5H8O | 84.1 | 750.5 | 187.05 | Dimer | 93.87 | 97.71 | 88.20 | 49.01 | 46.93 | 95.35 | 40.53 | 84.74 | 101.71 |
| ethyl 2-hydroxypropanoate | C97643 | C5H10O3 | 118.1 | 811.2 | 216.02 |  | 230.62 | 239.30 | 130.56 | 82.62 | 74.15 | 53.39 | 51.55 | 44.73 | 43.03 |
| Methyl isobutyl ketone | C108101 | C6H12O | 100.2 | 737.8 | 181.44 |  | 169.65 | 169.55 | 203.36 | 224.71 | 189.19 | 199.25 | 237.32 | 221.62 | 237.60 |
| methyl-5-hepten-2-one | C110930 | C8H14O | 126.2 | 992.2 | 344.54 |  | 29.51 | 33.15 | 28.26 | 27.72 | 27.87 | 30.89 | 34.23 | 29.52 | 33.11 |
| Pentanal-M | C110623 | C5H10O | 86.1 | 704.2 | 167.39 | Monomer | 285.90 | 287.46 | 325.57 | 348.04 | 379.84 | 337.75 | 342.93 | 335.20 | 363.36 |
| Pentanal-D | C110623 | C5H10O | 86.1 | 704.2 | 167.39 | Dimer | 412.81 | 416.93 | 366.96 | 327.85 | 366.89 | 433.02 | 468.52 | 489.11 | 560.19 |
| Ethyl Acetate-M | C141786 | C4H8O2 | 88.1 | 620.2 | 142.51 | Monomer | 1086.57 | 1085.31 | 1098.49 | 1145.71 | 1234.19 | 1128.19 | 1186.03 | 1169.08 | 1171.95 |
| Ethyl Acetate-D | C141786 | C4H8O2 | 88.1 | 614.2 | 140.99 | Dimer | 4391.40 | 4402.39 | 3495.47 | 3524.69 | 3609.40 | 4785.44 | 4478.90 | 4316.35 | 3822.67 |
| 2-Butanone-M | C78933 | C4H8O | 72.1 | 588.7 | 134.64 | Monomer | 272.07 | 281.28 | 304.53 | 303.09 | 295.93 | 283.59 | 287.53 | 290.44 | 298.86 |
| 2-Butanone-D | C78933 | C4H8O | 72.1 | 587.7 | 134.39 | Dimer | 437.47 | 419.34 | 460.12 | 433.91 | 375.23 | 481.34 | 413.02 | 479.32 | 463.46 |
| 1-propanol | C71238 | C3H8O | 60.1 | 561.9 | 128.29 |  | 766.19 | 784.27 | 743.77 | 651.16 | 590.50 | 722.57 | 738.45 | 749.18 | 703.39 |
| acetone | C67641 | C3H6O | 58.1 | 505.4 | 115.85 |  | 1581.95 | 1560.74 | 1357.50 | 1660.35 | 1580.60 | 1385.72 | 1389.33 | 1537.74 | 1163.74 |
| 1 | unidentified | * | 0 | 505.4 | 115.85 |  | 261.49 | 264.39 | 290.11 | 292.37 | 281.16 | 249.78 | 294.86 | 259.31 | 256.05 |
| 2-methylbutanal | C96173 | C5H10O | 86.1 | 668.4 | 155.46 |  | 36.82 | 40.35 | 43.48 | 63.11 | 61.20 | 57.93 | 46.57 | 53.36 | 49.14 |
| 3-methylbutanal-M | C590863 | C5H10O | 86.1 | 660.2 | 153.17 | Monomer | 72.75 | 76.85 | 81.41 | 70.69 | 88.24 | 66.70 | 108.69 | 107.35 | 112.10 |
| 2,3-pentanedione | C600146 | C5H8O2 | 100.1 | 696.6 | 164.35 |  | 131.36 | 135.95 | 149.21 | 159.62 | 175.94 | 136.59 | 168.32 | 149.51 | 153.39 |
| 2 | unidentified | * | 0 | 698.5 | 165.13 |  | 360.44 | 388.36 | 371.09 | 400.93 | 279.47 | 412.16 | 379.65 | 497.22 | 395.56 |
| methyl acetate | C79209 | C3H6O2 | 74.1 | 537.5 | 122.75 |  | 222.44 | 229.57 | 193.75 | 220.21 | 215.21 | 268.99 | 230.05 | 203.08 | 194.68 |
| pent-1-en-3-ol | C616251 | C5H10O | 86.1 | 682.5 | 159.47 |  | 818.51 | 820.20 | 831.94 | 828.77 | 801.30 | 836.65 | 793.97 | 823.69 | 836.75 |
| Propanoic acid | C79094 | C3H6O2 | 74.1 | 717.1 | 172.62 |  | 83.02 | 87.49 | 86.72 | 68.91 | 60.00 | 70.54 | 76.54 | 75.87 | 71.28 |
| 3 | unidentified | * | 0 | 578.8 | 132.25 |  | 71.40 | 73.32 | 65.30 | 59.30 | 48.62 | 52.23 | 98.51 | 67.11 | 56.04 |
| ethyl propanoate | C105373 | C5H10O2 | 102.1 | 713.8 | 171.30 |  | 46.10 | 47.26 | 40.70 | 42.36 | 42.54 | 44.62 | 52.89 | 47.44 | 40.34 |
| 4 | unidentified | * | 0 | 518.5 | 118.62 |  | 154.02 | 153.32 | 91.42 | 105.04 | 84.18 | 140.76 | 144.54 | 154.28 | 139.32 |
| Benzaldehyde-D | C100527 | C7H6O | 106.1 | 962.4 | 317.20 | Dimer | 26.68 | 33.45 | 33.12 | 26.93 | 29.82 | 45.71 | 31.30 | 42.76 | 86.54 |
| ( E)-2-hexenal-D | C6728263 | C6H10O | 98.1 | 847.7 | 235.26 | Dimer | 38.36 | 38.52 | 21.95 | 19.12 | 30.68 | 36.57 | 18.33 | 35.50 | 57.26 |
| 5 | unidentified | * | 0 | 880.1 | 253.86 |  | 24.51 | 25.72 | 42.76 | 44.30 | 34.00 | 37.14 | 44.38 | 50.54 | 58.69 |
| n-Hexanol | C111273 | C6H14O | 102.2 | 868.7 | 247.17 |  | 20.20 | 21.32 | 22.98 | 19.69 | 15.47 | 19.08 | 28.57 | 30.85 | 24.67 |
| pentan-1-ol-D | C71410 | C5H12O | 88.1 | 762.2 | 192.34 | Dimer | 32.07 | 35.43 | 32.75 | 25.82 | 22.88 | 31.41 | 35.40 | 51.00 | 53.92 |
| 2-Hexanone | C591786 | C6H12O | 100.2 | 780.9 | 201.17 |  | 47.89 | 50.59 | 62.76 | 67.43 | 60.84 | 62.52 | 80.64 | 75.62 | 79.79 |
| 3-methylbutanal-D | C590863 | C5H10O | 86.1 | 664.6 | 154.40 | Dimer | 29.83 | 36.20 | 37.71 | 51.67 | 66.09 | 46.23 | 53.95 | 53.44 | 60.18 |
| acetic acid | C64197 | C2H4O2 | 60.1 | 647.9 | 149.81 |  | 255.27 | 271.08 | 192.17 | 131.92 | 123.09 | 77.86 | 82.36 | 64.46 | 63.38 |
| 2-heptanone | C110430 | C7H14O | 114.2 | 889.4 | 259.40 |  | 112.64 | 114.15 | 108.20 | 118.53 | 111.81 | 150.31 | 166.67 | 181.93 | 201.57 |
| ethanol | C64175 | C2H6O | 46.1 | 439.4 | 102.84 |  | 1541.14 | 1622.42 | 1568.15 | 1898.19 | 1895.83 | 1887.92 | 1706.94 | 1360.93 | 1279.56 |

MW: molecular weight; RI: retention index; Rt: retention time.
